# [Retracted] MicroRNA‑381 serves as a prognostic factor and inhibits migration and invasion in non‑small cell lung cancer by targeting LRH‑1

**DOI:** 10.3892/or.2023.8640

**Published:** 2023-10-02

**Authors:** Chunyan Tian, Jun Li, Lili Ren, Ren Peng, Binbin Chen, Yumei Lin

Oncol Rep 38: 3071–3077, 2017; DOI: 10.3892/or.2017.5956

Following the publication of this paper, it was drawn to the Editor's attention by a concerned reader that certain of the western blotting data shown in Fig. 5C, and the cell migration and invasion data shown in Figs. 3C and D and 6B and C were strikingly similar to data that had already appeared in other articles. Owing to the fact that the contentious data in the above article had already been published prior to its submission to *Oncology Reports*, the Editor has decided that this paper should be retracted from the Journal. The authors were asked for an explanation to account for these concerns, but the Editorial Office did not receive a reply. The Editor apologizes to the readership for any inconvenience caused.

